# Chemical Modification of Agro-Industrial Waste-Based Bioadsorbents for Enhanced Removal of Zn(II) Ions from Aqueous Solutions

**DOI:** 10.3390/ma14092134

**Published:** 2021-04-22

**Authors:** David Castro, Nelly Ma. Rosas-Laverde, María Belén Aldás, Cristina E. Almeida-Naranjo, Víctor H. Guerrero, Alina Iuliana Pruna

**Affiliations:** 1 Department of Materials, Escuela Politécnica Nacional, Quito 170524, Ecuador; da.castro92@outlook.com (D.C.); victor.guerrero@epn.edu.ec (V.H.G.); 2 Department of Civil and Environmental Engineering, Escuela Politécnica Nacional, Quito 170524, Ecuador; maria.aldas@epn.edu.ec; 3 Department of Mechanical Engineering, Escuela Politécnica Nacional, Quito 170524, Ecuador; cristina.almeidan@epn.edu.ec; 4 Institute of Materials Technology, Universitat Politècnica de València, 46022 Valencia, Spain; 5 Center for Surface Science and Nanotechnology, University Politehnica of Bucharest, 313 Splaiul Independentei, 060042 Bucharest, Romania

**Keywords:** fruit peels, heavy metal removal, wastewater treatment, adsorption, kinetics

## Abstract

Contamination of water by heavy metals is a major environmental concern due to the potential ecological impact on human health and aquatic ecosystems. In this work, we studied the chemical modification of various fruit peels such as banana (BP), granadilla (GP), and orange ones (OP) in order to obtain novel bio-adsorbents to improve the removal of Zn(II) ions from 50 mg·L^−1^ synthetic aqueous solutions. For this purpose, sodium hydroxide and calcium acetate were employed to modify the fruit peels. The moisture, extractives, lignin, hemicellulose, and cellulose contents of the raw materials were determined according to ASTM standards. The obtained bio-adsorbents were characterized by scanning electron microscopy (SEM), Fourier Transform Infrared Spectroscopy (FTIR) and thermogravimetric analysis (TGA). The results showed the OP bio-adsorbents performed better, especially when the concentration of the modifier solutions increased, e.g., the OP particles modified using 0.8 M NaOH and Ca(CH_3_COO)_2_ solutions resulted in 97% removal of Zn(II) contaminating ions and reached a maximum adsorption capacity of 27.5 mg Zn per gram of bio-adsorbent. The adsorption processes were found to follow a pseudo-second order model. The error function sum of square error indicated the Freundlich isotherm (non-linear regression) as best fit model. The obtained results are particularly interesting for material selection in wastewater treatment technologies based on contaminant adsorption.

## 1. Introduction

Water contamination is one of the most important and high priority research topics around the world, as the availability of clean water is not only essential for life but it is also of paramount importance for social and economic development [1]. Unfortunately, as agricultural, industrial, and urban activities flourish, the pollution level in the environment and particularly in water bodies also increases [2,3]. Organic matter, nutrients, fertilizers, pesticides, dyes, pharmaceuticals, personal care products, oils, and heavy metals can be counted among the commonly found contaminants [4]. Furthermore, these organic and inorganic pollutions directly affect human health and aquatic ecosystems [5].

Particularly, contamination of water by heavy metals is worrisome because of their persistence and the possibilities of bioaccumulation and biomagnification [6,7]. These non-degradable and potentially toxic contaminants include arsenic, chromium, lead, cadmium, mercury, copper, and zinc, among others [8,9]. Their presence in water bodies is associated with natural and anthropogenic sources such as atmospheric precipitation, geological weathering, coal combustion, electroplating, leather tanning, and mining industry discharges [10]. The potential ecological impact of heavy metals and their effect on human health demand the development of effective treatment methods to remove them from water and wastewaters.

Zinc is one of the essential elements for life when it acts as a micronutrient. Nevertheless, in larger quantities, it is considered a toxic metal, and it was classified as such by the US Environmental Protection Agency [11]. Zinc can be used in several applications including protective layers for aluminum, in the galvanized steel industries, in the production of alloys, ceramics, rubbers, batteries, cosmetics and paints [12,13]. Due to vast industrial use, Zn(II) ion residues in wastewaters have been found to reach concentrations up to 54 mg·L^−1^ [10]. On the other hand, it has been defined that the concentration of Zn(II) ions in drinking water should be less than 5 mg·L^−1^ due to problems associated with its organoleptic properties as color, taste and odor [11]. Zn(II) ion concentrations greater than 15 mg·L^−1^ could cause health problems such as diarrhea, dehydration, abdominal pain, fever, nausea, damage to pancreas, anemia, and vomiting. Excessive exposure to, or intake of, zinc can deteriorate the liver [14]. Long-term exposure to high amounts of zinc can affect the brain, causing focal neuronal deficits and lethargy and can also increase the risk of prostate cancer [15]. Zn(II) is also related to Alzheimer’s disease [16]. Finally, its presence in rivers could cause bioaccumulation in aquatic organisms, indirectly affecting humans [17].

There are different methods to remove heavy metals from industrial wastewaters, including chemical precipitation, electrocoagulation, reverse osmosis, ion exchange, among others [18,19,20,21]. Nevertheless, most of them show disadvantages due to the technological requirements for installation, operation and maintenance, generation of toxic byproducts and high cost compared with methods such as adsorption [8,22,23,24]. Adsorption can be considered a promising alternative thanks to its relative simplicity, low cost, and high efficiency [25].

Activated carbon is one of the most widely used adsorbents for removing heavy metals from water [26,27]. However, the relatively high cost of materials such as this one has triggered the development of a variety of novel bio-adsorbents [28,29]. Bacteria (*Bacillus subtillis*, *Burkholderia cepacia*), fungi (*Rhizopus arrhizus*, *Hypholoma fasciculation*), yeasts (*Saccharomyces cerevisiae*), and algae are some of the alternatives used to eliminate Zn(II) ions [30,31].

However, relatively recent studies have also explored the possibility of removing Zn(II) ions by using agro-industrial residues such as orange peels, rapeseed residues, banana peel, dead biomass (*Variovax paradoxus* and *Arthrobacter viscousus)*, sugarcane bagasse, and lemon grass. These low-cost bio-adsorbents could reach Zn(II) ions removal efficiencies close to those of activated carbon (90% and higher) [32,33,34,35,36], which demonstrates the high potential of using agro-industrial residues for treating heavy metal contaminated waters.

Agro-industrial residues such as fruit peels contain hemicellulose, lignin, lipids, proteins, simple sugars, water, and pectin in their structure [37,38]. These components are involved in bio-adsorption processes due to their high content of carboxyl and hydroxyl groups [39]. However, in the absence of a prior treatment, agro-industrial residues could generate problems related to chemical and biological oxygen demand, organic carbon and a low adsorption capacity [40,41,42]. Therefore, for wastewater treatment, it is necessary to modify the agro-industrial residues to remove the soluble organic compounds and increase the adsorption capacity [43]. This is particularly relevant when these materials are used in wastewater treatment technologies based on adsorption (e.g., bio-filtration) [44]. Moreover, the material used as bed support not only plays a fundamental role in the contaminant removal but has an impact on the treatment cost [45].

In this work, we study the use fruit peels to obtain novel bio-adsorbents based on agro-industrial wastes for Zn(II) ion contaminants from synthetic wastewaters. The adsorption performance was studied upon chemical modification with sodium hydroxide and calcium salt, as an approach to improve the adsorption capacity. The adsorption performance of the bio-adsorbents was analyzed also with the starting waste peels, namely obtained from banana, granadilla, and orange peels. The Zn(II) ion removal efficiencies, adsorption capacity and contact time were determined as a function of the concentration of the base and salt solutions used for treatment.

## 2. Materials and Methods

### 2.1. Materials

Peels of banana (*Musa paradisiaca L*.) (BP), granadilla (*Passiflora ligularis*) (GP), and orange (*Citrus aurantium*) (OP) were used as raw materials for obtaining bio-adsorbents. They were collected from a local fruit market in Quito, Ecuador. Calcium acetate (Ca(CH_3_COO)_2_) and sodium hydroxide (NaOH), as well as zinc chloride (ZnCl_2_) and hydrochloric acid (HCl), were used for the peel modification and formulation of synthetic water solutions, respectively. The chemicals were reagent grade (Merck, Burlington, MA, USA) and used as received.

### 2.2. Bio-Adsorbent Preparation

The fruit peel wastes were washed with water in order to clean the surface from impurities [46] and further cut into pieces (2 × 2 cm^2^) and oven-dried at 60 °C for 24 h. Next, the dry peel pieces were crushed in a knife-mill (Thomas Wiley, model 3379-K05, Thomas Scientific, Swedesboro, NJ, USA) and sieved to a particle size between 125 and 841 µm.

To obtain the bio-adsorbents, the natural dye was removed by dispersing 30 g of fruit peel particles in 600 mL of water for 1 h. The peel particles were recovered from the supernatant by centrifugation at 1000 rpm for 20 min. This process was repeated until the supernatant got transparent. The peel particles were finally dried at 60 °C for 24 h. The bio-adsorbents prepared from banana, granadilla, and orange peels were named as BP, GP, and OP, respectively.

Further, a chemical modification of the peel particles was performed by treating them successively with NaOH and Ca(CH_3_COO)_2_ solutions of varying concentration each: 0.2, 0.5, and 0.8 M [34]. To this end, 30 g of peel particles were first added to 500 mL NaOH solution and stirred at 1000 rpm for 2 h. The peel particles were centrifuged and washed with water until supernatant reached pH 7 and further dried at 45 °C for 4 h. Next, 20 g of NaOH-treated fruit peel particles were added to 500 mL Ca(CH_3_COO)_2_ solution at pH 5 for 24 h. The pH of the mixtures was adjusted at pH 5 ± 0.5 using NaOH (1.0 N) or HCl (1.0 N) solutions. The modified peel particles were centrifuged and rinsed to remove the excess of calcium. This process was carried out at room temperature and repeated three times. Finally, peel particles were dried at 60 °C for 24 h [34,47]. The modified banana, granadilla, and orange peel bio-adsorbents were named as BP_x_, GP_x_, and OP_x_, respectively, where x is the concentration of the chemical solutions used for their modification, namely 0.2, 0.5, and 0.8 M.

### 2.3. Characterization

The moisture content of the BP, GP, and OP bio-adsorbents were determined according to ASTM D4442-16 (method A) and extractives, lignin, hemicellulose, and cellulose concentrations of dry samples were established through ASTM D1107-96 (2013) and ASTM D1106-13, respectively [48,49]. The thermal stability of the bio-adsorbents was evaluated by using thermogravimetry analysis (TGA) on a TGA Q500 (TA Instruments, New Castle, DE, USA) at a heating rate of 10 °C min^−1^ in a temperature range from 20 to 1000 °C in static air atmosphere [50,51]. The main functional groups of the unmodified and modified bio-adsorbents before and after metal uptake were identified by using Fourier transform infrared spectroscopy (FT-IR) on a Spectrum 200 spectrometer (Perkin Elmer, Waltham, MA, USA) within the 4000–500 cm^−1^ range, and the morphology of the bio-adsorbents was studied using a PSEM eXpress scanning electron microscope (SEM) (ASPEX, Delmont, PA, USA) working at 20 kV [50,51,52].

### 2.4. Adsorption Experiments

Batch bio-adsorption experiments were performed at room temperature to study the Zn(II) ion adsorption. Adsorption of Zn(II) ions was conducted using 1 g bio-adsorbent in 100 mL of 50 mg·L^−1^ ZnCl_2_ solution with magnetic stirring at 150 rpm for 240 min. The pH was measured using a pH meter (Mettler Toledo, FiveEas F20, Columbus, OH, USA) and it was adjusted to 5.0 ± 0.5 by adding either 0.1 mol·L^−1^ NaOH or 0.1 mol·L^−1^ HCl in all batch tests. After the adsorption, the suspension was filtered and the concentration Zn(II) ions in solution was determined by spectrophotometry analysis (HACH model DR 1900) (HACH, Loveland, CO, USA) using the Zincon method (Method 8009) [53,54]. All experiments were performed in triplicate.

Adsorption kinetics profiles were determined with a contact time from 0 to 4 h [34]. The removal data of Zn(II) ions using banana, granadilla, and orange peels chemically modified using 0.8 mol·L^−1^ solutions of NaOH and Ca(CH_3_COO)_2_ were fitted by pseudo-first model and by a pseudo-second order model, given by Equations (1) and (2) [55,56]:

(1) $$\log\left(q_{e}-{\;q}_{t}\right)=\log\left(q_{e}\right)-\frac{k_{1}}{2.303}t$$

(2) $$\frac{t}{q_{t}}=\frac{1}{k_{2}\times q_{e}^{2}}+\frac{t}{q_{e}},$$

where k_1_ (min^−1^) is the kinetics constant of pseudo-first order, k_2_ is the kinetics constant of pseudo-second order (g bio-adsorbent/mg Zn/min); q_e_ is the capacity of biosorption in equilibrium (mg Zn/g bio-adsorbent) and q_t_ is the biosorption capacity (mg Zn/g bio-adsorbent) at time t (min). The values of k_2_ and q_e_ allow to determine the initial velocity of adsorption h (mg Zn/g adsorbent/min), which is given by Equation (3):

(3) $$h=k_{2}\times q_{e}^{2},$$

To obtain k_2_ and q_e_, t/q_t_ were analyzed as a function of time (t). Adsorption isotherms were obtained by adding 1 g of peel particles to 100 mL of Zn(II) solutions with concentrations between 25 and 350 mg·L^−1^, at pH 5.0 ± 0.5, during the equilibrium time [50]. Further, the obtained data was fitted to isotherm models, namely Freundlich and Langmuir by using the linearized forms in Equations (4) and (5) [50,57] and the non-linear forms in Equations (6) and (7) [58]. The adsorption model with the best fit was indicated by the highest correlation coefficient.

(4) $$\log q_{e}=\frac{1}{n}\log C_{e}+\log K_{F}$$

(5) $$\frac{C_{e}}{q_{e}}=\frac{1}{\left(q_{m}\times K_{L}\right)}+\frac{1}{q_{m}}{\;C}_{e}$$

(6) $$q_{e}=K_{F}{\;C}_{e}{}^{1/n}$$

(7) $$q_{e}=\frac{C_{e}{\;q}_{m}\;K_{L}}{1+K_{L}C_{e}}$$

where q_e_ is the amount of solute adsorbed at equilibrium per adsorbent unit weight (mg·g^−1^), C_e_ represents the concentration of solute at equilibrium (mg·L^−1^), K_F_ is the constant of the Freundlich equation related to adsorption capacity (L ^1/n^·mg^1−1/n^ g^−1^), *n* is the coefficient of the Freundlich equation related to heterogeneity, and q_m_ represents the maximum monolayer capacity (mg·g^−1^) while K_L_ is the empirical constant of the Langmuir equation (L·mg^−1^). The separation factor or equilibrium parameter (R_L_) is a dimensionless constant and a fundamental characteristic of the Langmuir isotherm. This constant is defined according to Equation (8) [59], where K_L_ is the Langmuir constant (L·mg^−1^) and is associated with the adsorption energy, and C_o_ is the initial concentration (mg Zn L^−1^). R_L_ is indicative of the isotherm shape and the nature of the adsorption, that is, when R_L_ = 0, the adsorption is reversible, when 0 < R_L_ < 1, then the adsorption is favored, if R_L_ = 1 the adsorption is linear and when R_L_ > 1, the adsorption is not favored [59]:

(8) $$R_{L}=\frac{1}{\left(1+{\;K}_{L}\times C_{o}\right)},$$

The data were fit to these kinetic and isotherm models because they are the classic models and have shown to adjust well to the equilibrium models (R^2^ ≈ 1), described by bio-adsorbents residues in the removal of several contaminants [60]. The data of the linear isotherm models was calculated and compared to that computed (Microsoft Excel SOLVER software, version 2016, Quito, Ecuador) for non-linear isotherm models, taking into account the sum of squares error (SSE) as error function which is necessary for optimization of the fitting [58].

## 3. Results and Discussion

Compounds such as cellulose, hemicellulose, lignin, glucose, galactose, arabinose, rhamnose, and xylose are involved in bio-adsorption processes as they have carboxyl and hydroxyl functional groups [27,28,31,35,39,61,62]. Table 1 shows moisture content and extractives, as well as lignin, hemicellulose, and cellulose content of unmodified banana, granadilla, and orange peels. These values are expressed as a percentage of the initial dry weight of materials [63]. As it can be observed in Table 1, the moisture contents for banana, granadilla, and orange peels are similar to those reported elsewhere [31,35,61,64]. The banana peels exhibit the highest moisture content (89.1%) which could be due to some factors such as structural differences, genetic varieties, agronomic, and climatic conditions during growth [64,65].

Lignin, hemicellulose, and cellulose are known to exhibit great sorption capacity of some pollutants [66]. Lignin is an organic polymer with polar functional groups that could bond chemically and interact with pollutant cations [66]. As can be observed in Table 1, banana and granadilla bio-adsorbents show similar lignin contents, while orange peels show the lowest value, in agreement with other studies on banana peels [67,68] and orange peels [39,69]. On the other hand, granadilla peels are compared with passion fruit peels since these fruits belong to the same family [70] and showed lower lignin percentages than the values previously reported for yellow passion fruit peels [71]. Among other factors, this difference can be attributed to the stage of ripeness of the studied fruits [38].

Cellulose is a polymer with a large quantity of hydroxyl groups, bonded to hemicellulose by hydrogen bonds. This structure shows a highly reactive surface area which can adsorb cations. Therefore, adsorption capacity is increased when cellulose content is higher [72]. Banana dry peels show the highest cellulose percentage (62.5%), compared to other values reported [46,67,68,73]. This is also the case for orange dry peels (55.7%) [39], while granadilla dry peels exhibit the lowest (24.6%).

On the other hand, hemicellulose percentages of banana and orange peels are similar as reported elsewhere [39,46,67,68,73]. For yellow passion fruit peels, the hemicellulose and cellulose contents are of 39 and 26%, respectively [74]. Factors such as the conditions in which the fruit was collected, type of species, state of ripeness of the fruit, conditions of cultivation, and the methods used in the peels characterization could explain this difference [75].

Extractives can be considered as phenolic substances which can interact with the contaminants [69]. The presence of extractives reduces the adsorption capacity of materials since these substances could fill the pores of the absorbent, preventing the adsorbate molecules from interacting with the active sites. Hence, a material with a high content of extractives shows a low adsorption capacity [76]. Granadilla peels exhibit the highest percentage of extractives, similar to that of yellow passion fruit peels [74]. On the other hand, banana peels showed the lowest value. Nevertheless, those results are quite different from those reported for banana and orange peels by previous authors [68,69].

### 3.1. Characterization of the Adsorbents

**Figure 1:**
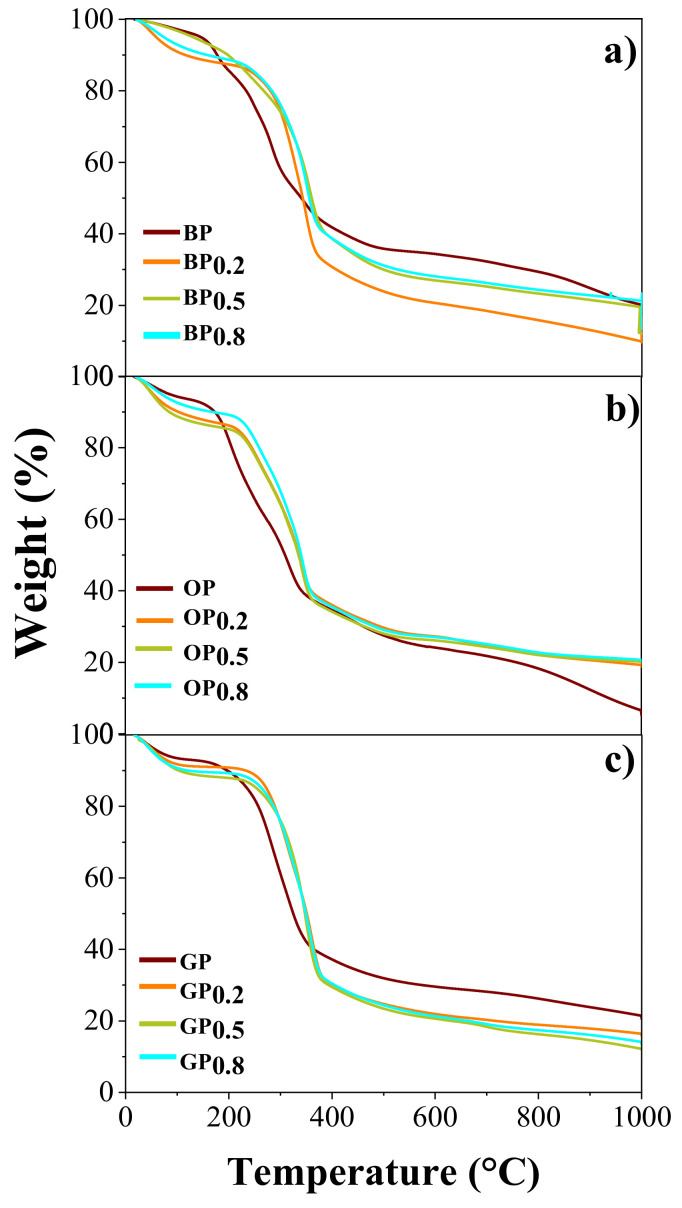
Thermogravimetric analysis profiles of (**a**) Banana (BP), (**b**) Orange (OP), and (**c**) Granadilla (GP) unmodified and chemically modified peels.

Thermogravimetric analysis (TGA) was carried out on unmodified and modified bio-adsorbents which were previously dried. Figure 1 shows the decomposition according to the thermal profiles.

As shown in Figure 1, unmodified bio-adsorbents show a first weight loss of about 2.5, 5.2, and 8.2% for banana, granadilla, and orange peels, respectively, which was recorded at 70–100 °C, and it is attributed to the evaporation of water [77]. Another clear weight loss was observed above 220–250 °C, which corresponds to the degradation of hemicellulose and organic and/or aqueous extracts such as pectin, which represent the reduction of the volatile matter [77]. The next stage, between 390 and 400 °C, represents the degradation of the cellulose found in each peel. The last stage corresponds to the degradation of lignin that occurs at temperatures above 400 °C [69].

The thermograms of the modified peels show a slight right shift in all the stages with respect to unmodified peels thermograms, which could be due to the presence of calcium in the peel structure because of the chemical treatment carried out [55]. Due to its resistance to temperature, calcium (melting temperature 842 °C) and its compounds act as thermal stabilizers of bio-adsorbents, this effect has also been observed when they are mixed with synthetic polymers [78].

**Figure 2:**
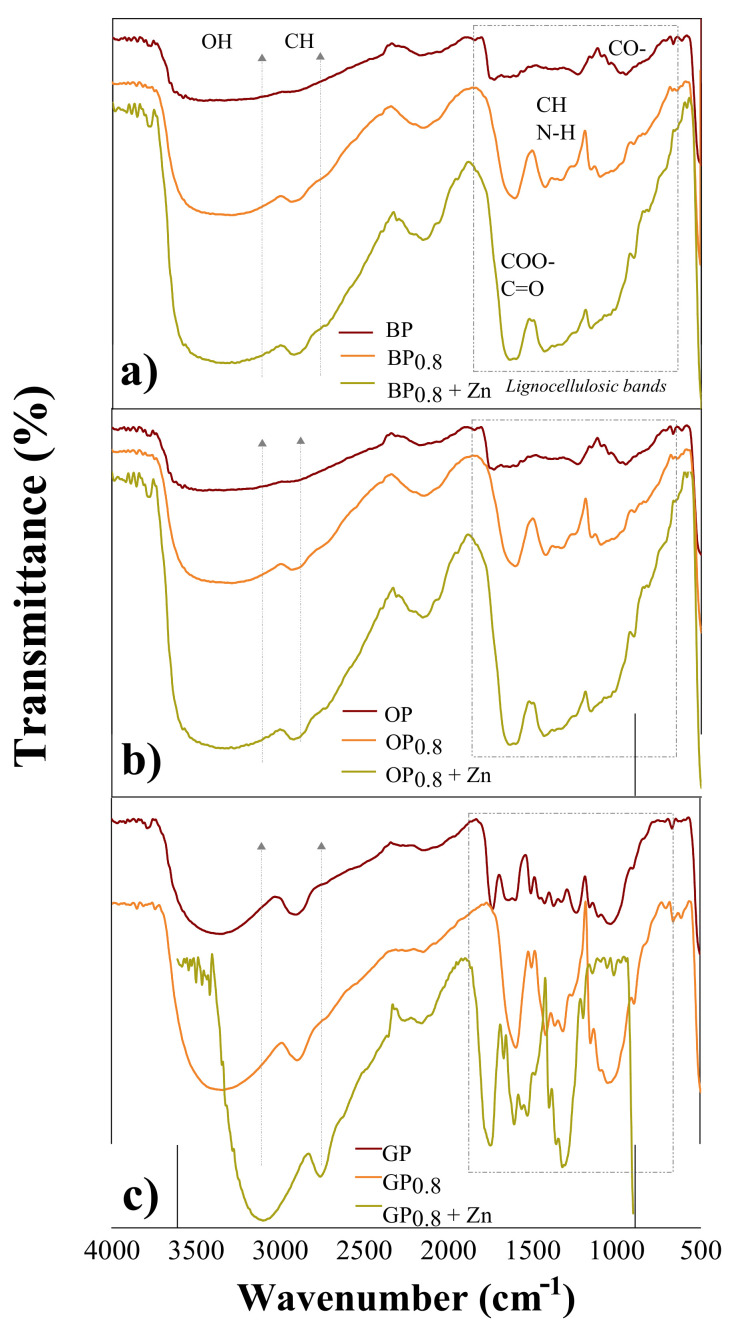
FT-IR spectra for (**a**) banana (BP), (**b**) orange (OP), and (**c**) granadilla (GP) bio-adsorbents before and after chemical treatment and the Zn(II) ion removal.

Figure 2 shows FT-IR spectra obtained from the unmodified and the chemically modified bio-adsorbents, before and after the adsorption of Zn(II) ions. For exemplification, the spectra of adsorbents treated with 0.8 M modifiers are presented.

As shown in Figure 2, banana, granadilla, and orange bio-adsorbent spectra show bands between 3600–3000 cm^−1^, which correspond to the hydroxyl group (OH^–^). These bands are due to the stretching of the OH^–^ bonds of cellulose, hemicellulose, lignin, citric acid, and water from the residual moisture of the peels [79]. The bands located between 3000 and 2800 cm^−1^ belong to the stretching of C–H groups. The bands between 1744 and 1638 cm^−1^ correspond to the carboxylate ion (COO^–^) and the carbonyl ester group (C=O) stretching from aldehydes and ketones. The bands within the 1430–1455 cm^−1^ range indicate aliphatic and aromatic groups (C–H) associated to the in-plane vibrations from methyl, methylene, and methoxy groups. The bands around 1422–1420 cm^−1^ were assigned to N–H bending vibrations. The bands located withing 1300–1000 cm^−1^ range are assigned to the stretching vibration of carboxylic groups (C–O) from acids and alcohols. The identified groups are present in hemicellulose (1246 cm^−1^), lignin (1518 cm^−1^), lipids (2927 cm^−1^), and pectin (1740 cm^−1^), principal peel components [80,81]. The O–H, N–H, C–O, and COO^–^ groups participate in sorption processes, being important sites for the pollutants location [82,83].

Upon chemical modification with 0.8 M modifiers, the bio-adsorbents spectra show changes in the bands located between 1700 and 1000 cm^−1^, which can be explained by the pre-treatment with NaOH, which caused the degradation of the organic compounds (hemicellulose, lignin, lipids, and pectin) and generated the methyl ester group hydrolysis and the ester groups transformation into carboxylate ions [55,82]. This treatment solubilizes some compounds that could interfere with the adsorption process, changes pore structure and increases the surface area of peels. Additionally, OH^–^ functional groups are incorporated during the basic activation [83,84]. This is evidenced by the increase of the bands between 1000 and 1700 cm^−1^ in the three bio-adsorbents (orange lines, Figure 2), and this is due to the high affinity of calcium ions for the carboxyl and amino groups present in the lignocellulosic groups [85]. The spectra of the modified bio-adsorbents after Zn(II) ion removal process show carboxylic (C–O), carbonyl ester (C=O), and hydroxyl (OH^–^) groups located at a shorter wavelength than before the removal process. The displacement associated to these groups is related to their participation in the adsorption of Zn(II) ions, specifically for OH^–^ groups. This indicates that the adsorption of the metal occurs on the bio-adsorbent surface [55,86].

**Figure 3:**
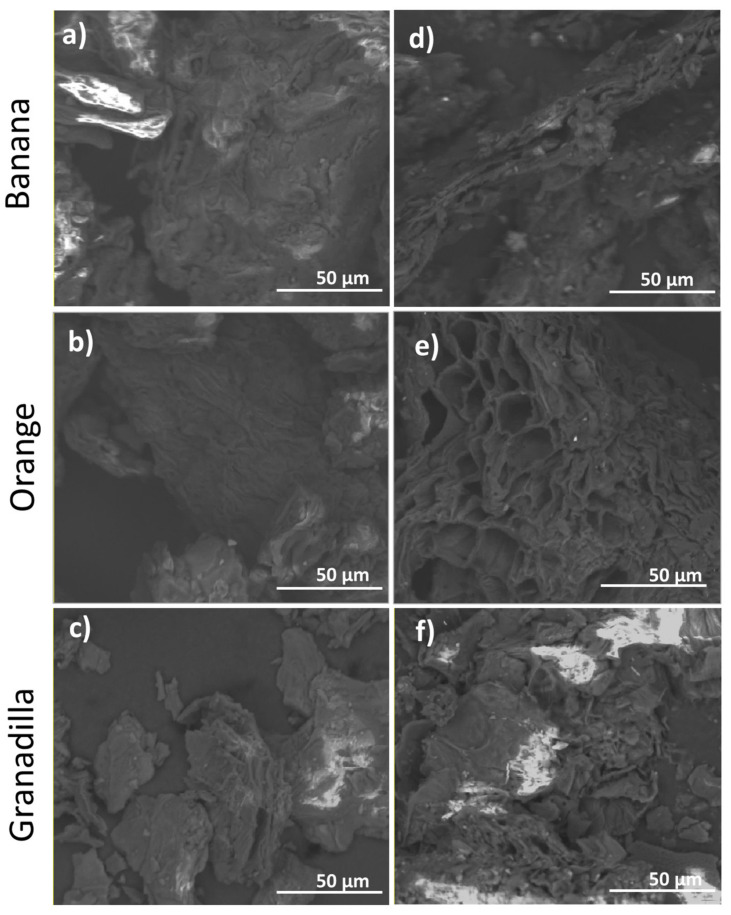
SEM micrographs of banana, orange, and granadilla peel adsorbents before (**a**, **b** and **c**, respectively) and after the chemical treatment with 0.8 M modifiers (**d**, **e**, and **f**, respectively).

Figure 3 shows the peel adsorbents before and after chemical treatment (BP, OP, GP, and BP_0.8_, OP_0.8_, GP_0.8_, respectively). The micrographs of non-treated bio-adsorbents showed an irregular morphology. However, upon chemical treatment, the BP_0.8_, OP_0.8_, and GP_0.8_ peel particles showed a porous structure due to the removal of soluble substances in the basic media treatment. It can be clearly seen that the porosity is the best in case of OP_0.8_ bioadsorbent. This treatment would generate a greater specific surface area that could contribute to Zn adsorption [84,87]. These modified particles further showed a rougher and less porous surface upon the metal ions removal because the ions they have partially covered the pores of the peel structure [84,88].

### 3.2. Influence of Chemical Treatment on Zinc Adsorption

The biosorption mechanisms depend on the metal or compound to be removed (adsorbate) and the organic material used as adsorbent (bio-adsorbent). These mechanisms include: (i) physical adsorption, where metal molecules are linked to the adsorbent surface due to electrostatic attraction forces, (ii) ion exchange (chemisorption), producing interchange between carboxyl, hydroxyl, amino, sulfonic groups and metal ions, and (iii) complexation, in which the union between the organic material and the metal is given by the formation of complexes on the material surface [17].

Table 2 shows the influence of the chemical treatments on the Zn(II) ion adsorption. As it can be observed, the adsorption efficiency of all fruit peel adsorbents increases upon chemical treatment. The best efficiency was recorded for the 0.8 M concentration of the NaOH and Ca(CH_3_COO)_2_ modifier solutions. The highest removal percentages were 92.57 ± 0.24, 97.13 ± 0.21, and 88.21 ± 0.34% for the banana, orange, and granadilla peel particles modified using 0.8 mol·L^−1^ solutions. The adsorption increase could be attributed to the combined changes introduced by the chemical treatment, in agreement with Feng and Guo [34], considering factors such as: the modification of the peel surface in such a way so as to favor the physical adsorption. Thus, in agreement with SEM images, the adsorbent with best porosity (orange) exhibited the best performance. Furthermore, in agreement with FT-IR spectra, the carboxylate groups obtained by transformation of methyl ester groups are known to have a greater capacity for binding with metal ions which favors ion exchange during the adsorption process, so the calcium ions attached to the pectin chains are exchanged with the metal ions. The ion exchange could cause the increase of the bands between 1000 and 1700 and around 3000 cm^−1^, this is probably because zinc is heavier than calcium. Similar behavior was observed in a research in which pectinate gels were modified with calcium and zinc ions [85,89,90]. On the other hand, the higher content of cellulose in the banana and orange bio-adsorbents (see Table 1) could be accounted for their better removal performance with respect to granadilla adsorbent [34].

### 3.3. Adsorption Kinetics

**Figure 4:**
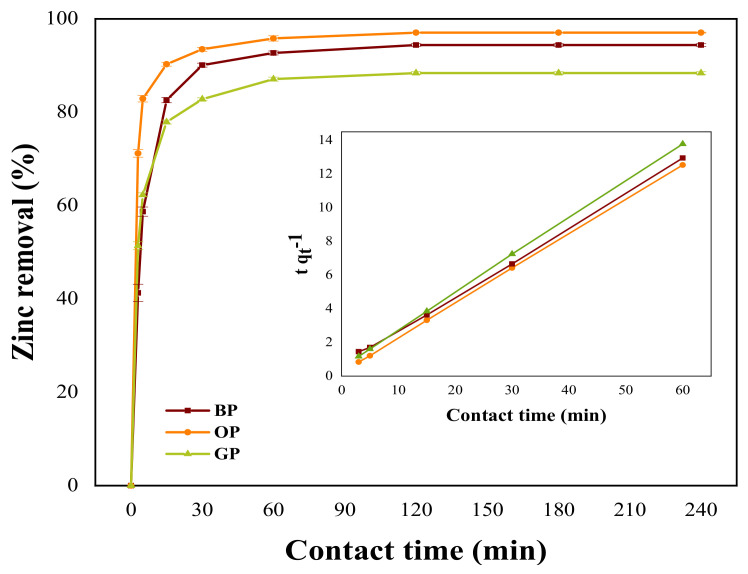
Kinetic profile of Zn(II) ion adsorption using the modified BP_0.8_, OP_0.8_, and GP_0.8_ peel bio-adsorbents. The inset shows the kinetics parameters.

Figure 4 shows the adsorption kinetic profiles for the removal of Zn(II) ions using the modified BP_0.8_, GP_0.8_, and OP_0.8_ peel adsorbents. These materials were chosen for the kinetics and isotherm studies because they are the ones that show the highest removal efficiencies. As can be observed, as the contact time increases, the Zn(II) ion removal also increases.

The adsorption process can be divided in two zones: in the first zone (corresponding to the first 20 min), the adsorption was very rapid as the bio-adsorbent surface was unsaturated and the active (or binding) sites are available. The binding sites are the places that receive the metal ions from the synthetic solution and where an ion exchange or a physical adsorption can occur [58,91]. In the second zone, the adsorption was slow due to the diffusion of Zn(II) ions to the internal part of the bio-adsorbent. At this stage, the equilibrium was reached for a contact time of 120 min. The equilibrium time was used to obtain the adsorption isotherms.

The kinetics of the removal of Zn(II) ions using banana, granadilla, and orange peels chemically modified using 0.8 mol·L^−1^ solutions of NaOH and Ca(CH_3_COO)_2_ was fitted by both pseudo-first order model and a pseudo-second order model. The results of fitting are presented in Table 3. By comparing the correlation coefficients, it can be observed that those of pseudo-second order model fit better the processes with each adsorbent considered. This indicates that Zn biosorption velocity for each peel is controlled by chemical adsorption [55], which is also supported by the results of the adsorbent characterization (presence of active sites/functional groups).

The values of q_e_ show that the greater capacity of removal in equilibrium is achieved using the chemically modified OP_0.8_ peel bio-adsorbent, which is consistent with the results presented in the previous section. The values of initial velocity of adsorption (h) show that the OP_0.8_ bio-adsorbent has the highest initial velocity for the Zn(II) ion adsorption. According to Sirilamduan et al., a greater presence of binding sites on the peel surface would favor the first stage of adsorption [91]. The greatest presence of binding sites is the result of the composition and structure of the peels and the chemical treatment received.

### 3.4. Adsorption Isotherms

The obtained results were fitted to the linear forms of Langmuir and Freundlich models. Table 4 shows the parameters calculated for each bio-adsorbent. According to the correlation coefficients, the removal of Zn(II) ions using chemically modified bio-adsorbents fits the Langmuir model for all the bio-adsorbents, which is indicative of an adsorption process given by a monolayer adsorption on the peel surface. According to Dada et al., this is due to the presence of many binding sites with similar adsorption energies at the bio-adsorbent surface [92]. The adsorption isotherms (obtained by using the linear forms) for the removal of Zn(II) ions using the modified BP_0.8_, GP_0.8_ and OP_0.8_ bio-adsorbents were obtained, where the individual points of each isotherm represented the experimental data obtained from the equilibrium concentrations (C_e_), whereas the trend lines represented the isotherm obtained using the Langmuir constant (K_L_) and the maximum adsorption capacity (q_m_). It was observed that the biosorption capacity in equilibrium (q_e_) at low equilibrium concentrations (<75 mg Zn L^−1^) increased, while at high equilibrium concentrations it tended to be constant [34]. The values of C_o_ between 25 and 350 mg Zn L^−1^ resulted in separation factor R_L_ values of 0.058–0.464, 0.055–0.45, and 0.028–0.286 for banana, orange, and granadilla peel adsorbents, respectively. These results show that adsorption is favorable for each type of peel (R_L_ < 1) [93]. In addition, the values of n, coefficient of the Freundlich equation related to heterogeneity, can describe the condition of adsorption (good adsorption if 2 < n < 10, poor adsorption if 1 < n < 2, and difficult adsorption if n < 1). The results in the Table 4 show good adsorption of Zn(II) in the surface of BP_0.8_ and OP_0.8_ and poor adsorption in the surface of GP_0.8_. However, according to the correlation coefficients, the removal of Zn(II) ions using chemically modified bio-adsorbents fits the Langmuir model [21].

**Figure 5:**
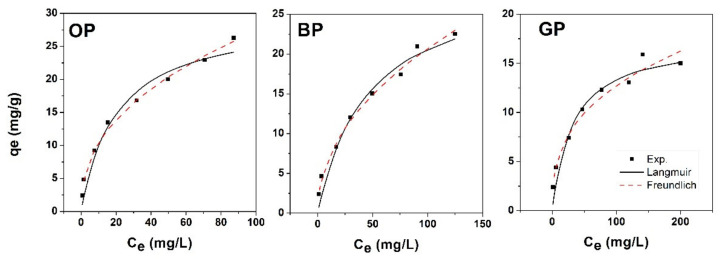
Zn(II) ion adsorption isotherms using chemically modified BP_0.8_, GP_0.8_, and OP_0.8_ peel adsorbents.

The adsorption of Zn(II) on the fruit peel bio-adsorbents was also analyzed with non-linear isotherm models as presented in Figure 5. The correlation coefficients are close to 0.9, suggesting the acceptability of both linear and non-linear models. However, given that linear models might introduce errors during data transformation, one could consider the non-linear fitting as more appropriate. The error function SSE was employed for the best fit model and it varied considerably although the non-linear correlation coefficients were similar. Thus, lower SSE was obtained for Freundlich isotherms for all bio-adsorbents, which confirmed the fitness of the model.

Table 5 compares the adsorption capacities, q_m_, of the bio-adsorbents studied in this work and some bio-adsorbents previously studied in literature and obtained using different peels with and without treatment. As it can be observed, in several cases the adsorption capacities of BP_0.8_ and OP_0.8_ peel bio-adsorbents were higher than other reported bio-adsorbents which is indicative of the significant potential the treatment with NaOH and Ca(CH_3_COO)_2_ have on the bio-adsorbents efficiency a for removing Zn(II) ions from aqueous solutions.

## 4. Conclusions

Agro-industrial waste based bio-adsorbents were obtained from fruit peels to be investigated for Zn(II) ion removal from synthetic waters. The effect of the starting waste material was investigated, namely, the adsorbent was obtained from three types of fruit peels such as based on banana, orange, and granadilla peels. In order to improve the adsorption performance of the bio-adsorbents, a chemical treatment with NaOH and Ca(CH_3_OO)_2_ was applied to remove them. The results indicate the efficiency of the obtained bio-adsorbents increases as the concentration of NaOH and Ca(CH_3_OO)_2_ employed for chemical modification also increases. The highest Zn(II) ion removal efficiencies achieved using BP_0.8_, OP_0.8_, GP_0.8_ peels were 92.57, 97.13, and 88.21%, respectively. The adsorption kinetics follow a model of pseudo-second order with equilibrium biosorption capacities for each peel of 4.79, 4.88, and 4.46 mg Zn/g bio-adsorbent and an equilibrium time of 2 h, while the Langmuir model is the one that best fits the behavior of the peels, with the maximum adsorption capacities being 25.59, 27.48, and 16.61 mg Zn g^−1^ bio-adsorbent for banana, orange, and granadilla peels, respectively. Although the adsorption mechanism with such sorbents is a complex matter, the improved removal performance could be attributed to the greater presence of binding sites as a result of the composition and structure of the peels and the chemical treatment received. In the case of banana and orange-based bio-adsorbents, their higher content of cellulose with respect to granadilla one could be taken into account. The bio-adsorbents obtained from modified banana, granadilla, and orange peels represent inexpensive materials that could be used in the removal of heavy metal from contaminated effluents and constitutes a new green technology that takes advantage of agro-industrial residues. This work indicates chemically modified agro-industrial residues could also be used in treatment technologies, in which adsorption is one of the main mechanisms for pollutant removal.

## Figures and Tables

**Table 1:** Physical and chemical characteristics of banana, orange, and granadilla peels.

Parameter (%)	Banana Peels	Orange Peels	Granadilla Peels
Lignin *	16.6 ± 1.1	7.9 ± 0.2	16.9 ± 0.6
Hemicellulose *	15.9 ± 0.1	15.4 ± 0.3	10.1 ± 0.7
Cellulose *	62.5 ± 1.0	55.7 ± 0.9	24.6 ± 1.3
Ethanol-toluene extractives *	4.7 ± 0.6	21.1 ± 1.2	48.5 ± 0.7
Moisture	89.1 ± 0.3	68.7 ± 0.4	68.8 ± 0.5

* Dry basis.

**Table 2:** Zn removal percentage achieved with bio-adsorbents.

Adsorbent	Zn Removal (%)	Adsorbent	Zn Removal (%)	Adsorbent	Zn Removal (%)
BP	85.5 ± 0.5	OP	91.9 ± 0.1	GP	74.5 ± 1.1
BP_0.2_	87.4 ± 0.2	OP_0.2_	93.4 ± 0.1	GP_0.2_	83.1 ± 0.8
BP_0.5_	90.2 ± 0.3	OP_0.5_	96.2 ± 0.3	GP_0.5_	85.6 ± 0.2
BP_0.8_	92.6 ± 0.2	OP_0.8_	97.1 ± 0.2	GP_0.8_	88.2 ± 0.4

**Table 3:** Kinetics parameters for Zn(II) ions removal using the chemically modified BP_0.8_, GP_0.8_, and OP_0.8_ bio-adsorbents.

Peel Adsorbents	Pseudo First Order			Pseudo Second Order			
	$q_{e}$ $\left[\frac{mg\;Zn}{g}\right]$	$k_{1}$ $\left[min^{-1}\right]$	R^2^	$q_{e}$ $\left[\frac{mg\;Zn}{g}\right]$	$k_{2}$ $\left[\frac{g}{mg\;Zn\times min}\right]$	h $\left[\frac{mg\;Zn}{g\times min}\right]$	R^2^
BP_0.8_	2.65	0.058	0.9145	4.81	0.09	2.06	0.9997
OP_0.8_	0.96	0.049	0.9303	4.83	0.27	6.49	0.9999
GP_0.8_	1.67	0.056	0.9720	4.44	0.14	2.73	0.9998

**Table 4:** Linear and non-linear adsorption isotherm parameters for the Zn(II) ions removal using the chemically modified BP_0.8_, GP_0.8_, and OP_0.8_ peel adsorbents.

Isotherm	BP_0.8_	OP_0.8_	GP_0.8_
Linear Langmuir			
qm (mg/g)	25.59	27.48	16.61
K_L_ (L/mg)	0.046	0.099	0.049
R^2^	0.9925	0.9945	0.9949
Linear Freundlich			
KF (mg/g) (L/mg)1/n	2.14	3.72	1.97
n	1.94	2.16	2.42
R^2^	0.9738	0.9679	0.9763
Non-Linear Langmuir			
qm (mg/g)	29.25	29.12	17.28
K_L_ (L/mg)	0.024	0.055	0.034
SSE	12.47	19.11	9.42
R^2^	0.9932	0.9950	0.9945
Non-Linear Freundlich			
KF (mg/g) (L/mg)1/n	2.41	3.99	2.56
n	2.14	2.39	2.86
SSE	2.87	2.85	5.30
R^2^	0.9925	0.9990	0.9940

**Table 5:** Adsorption capacities of various bio-adsorbents.

Adsorbent	Modification	Adsorbent Dose (g L^−1^)	Contact Time (h)	pH	$q_{m}(\mathrm{mg}\;\mathrm{Zn}\;g{-}^{1})$	Ref.
Mimusops Elengi	without	1	1.5	5	16.39	[94]
Rapeseed waste	without	1	24	5	13.86	[32]
Durian	Acid-treated HCl	5	4	8	36.75	[58]
BP_0.8_	NaOH and Ca(CH_3_COO)_2_	1	2	5	25.59	This work
Orange	without	4	3	5	33.08	[95]
OP_0.8_	NaOH and Ca(CH_3_COO)_2_	1	2	5	27.48	This work
GP_0.8_	NaOH and Ca(CH_3_COO)_2_	1	2	5	16.61	This work

## Data Availability

The data presented in this study are available on request from the corresponding author.
